# Synthesis and Characterization of Fatty Acid Grafted Chitosan Polymeric Micelles for Improved Gene Delivery of VGF to the Brain through Intranasal Route

**DOI:** 10.3390/biomedicines10020493

**Published:** 2022-02-19

**Authors:** Richard Nii Lante Lamptey, Avinash Gothwal, Riddhi Trivedi, Sanjay Arora, Jagdish Singh

**Affiliations:** Department of Pharmaceutical Sciences, School of Pharmacy, College of Health Professions, North Dakota State University, Fargo, ND 58105, USA; richard.lamptey@ndsu.edu (R.N.L.L.); avinash.gothwal@ndsu.edu (A.G.); riddhi.trivedi@ndsu.edu (R.T.); or sanjay.arora@ndus.edu (S.A.)

**Keywords:** intranasal, chitosan grafted micelles, blood–brain barrier, transfection, polymer, synthesis, targeted gene delivery, polyplex

## Abstract

Multifunctional fatty acid grafted polymeric micelles are an effective and promising approach for drug and gene delivery to the brain. An alternative approach to bypass the blood–brain barrier is administration through intranasal route. Multifunctional fatty acid grafted polymeric micelles were prepared and characterized for pVGF delivery to the brain. In vitro pVGF expression was analyzed in bEnd.3 cells, primary astrocytes, and neurons. Comparative in-vivo pVGF expression was analyzed to evaluate the effective route of administration between intranasal and intravenous. Biocompatible, multifunctional polymeric micelles were prepared, having an average size of 200 nm, and cationic zeta potential. Modified polymers were found to be hemo- and cyto-compatible. When transfected with the different modified chitosan formulations, significantly (*p* < 0.05) higher VGF expression was observed in primary astrocytes and neurons using the mannose, Tat peptide, and oleic acid grafted chitosan polymer. Compared to intravenous administration, intranasal administration of pVGF in polyplex formulation led to significantly (*p* < 0.05) higher pVGF expression. Developed multifunctional polymeric micelles were an effective pVGF delivery platform to the brain. Mannose and Tat ligand tagging improved the pVGF delivery to the brain.

## 1. Introduction

Safe drug and gene delivery to the brain persists as a major challenge, even in the presence of vast research in this field [1]. This is largely due to the protective effect of the blood–brain barrier (BBB) [2,3]. This restrictive property of the BBB keeps greater than 99% of potential therapeutics for the management of neurological disorders out of the brain [4]. Additionally, most drugs can not readily cross the BBB due to their pharmacokinetic profile [5]. Owing to this, brain-targeted delivery strategies have gained traction over the years. More recent brain targeting techniques rely on disruption of the BBB, and therefore have seen very little translation into clinical settings [6].

The human brain is situated right above the upper regions of the nostrils, only separated by a sieve-like structure (cribriform plate) [7]. This situation makes the brain accessible for targeted delivery through the nostrils. Although, the nasal epithelial presents as a barrier over the plate, the tightness of this barrier is low due to the leaky nature of the epithelial tissues [8]. Further, the brain and nose are directly connected by nerves which begin from the upper portions of the nostrils and end in the brain. The olfactory nerve is one of these nerves [9]. Drug delivery through the intranasal route involves the passage of drug formulations through the nostrils to achieve a certain pharmacological effect. The intranasal pathway presents a non-invasive administration route for pharmaceutical agents for local, systemic, and central nervous system (CNS) action [4]. Several studies have shown a direct route of transport from the olfactory region to the central nervous system in animal models, without prior absorption to the circulating blood [5].

The intranasal route can prove incredibly useful with aided drug delivery [10]. More recently, nanoparticles have been used to achieve targeted drug delivery; mostly after functionalization of these particles [11,12,13,14]. Specifically, chitosan-based vectors have received great attention over the past decade [15,16,17,18]. Interest in chitosan-based vectors is mostly due to the ease of modification, biocompatibility, and biodegradability [19,20,21]. Although a promising delivery tool, gene transfections achieved with unmodified chitosan are not desirable [19]. However, the pk/pd properties of chitosan nanomicelles have been widely improved by functionalization. Chemical modifications that employ the hydroxyl and primary amine groups have often resulted in nanoparticles with highly improved pk/pd properties of chitosan [19,22,23].

Cell penetrating peptides have shown great potential in improving brain uptake through the intranasal route. Several studies involving peptide conjugated to chitosan have also concluded in superior transfections compared to unmodified chitosan [15,17,24,25,26]. Further, the introduction of hydrophobic groups by grafting fatty acid into chitosan is known to lead to superior transfections. Studies show HIV-_1_ Transactivator of transcription (TAT) peptide aids direct translocation and endocytosis processes [27,28]. The grafting of TAT peptide to chitosan can improve the membrane permeability and result in products with improved transfection. Improvement of gene transfection with the dual-modified polymers will be due to an improved translocation and effective dissociation to release free plasmid deoxyribonucleic acid (pDNA) intracellularly. Unfortunately, very little research has been conducted considering the vast potential of peptide conjugated nanomicelles. This situation has left a gap in nanomicelle science that needs to be filled.

This research focuses on targeted nerve growth factor-inducible VGF (non-acronymic) gene delivery to the brain through the intranasal route. VGF plays a critical role in learning and memory, pathophysiology of psychiatric disorders, and neurodegenerative diseases [29]. Indeed, several animal studies have shown that forced expression of VGF gene and VGF-derived peptides attenuate Alzheimer’s disease (AD)-related phenotypes [29,30,31,32]. Therefore, the purpose of this study is to develop a novel effective gene therapy for AD by targeting VGF through the intranasal route. The objective of this study is also to compare the gene transfection achieved with modified chitosan nanomicelles when delivered both intranasally and intravenously. To achieve our goal, chitosan was modified with oleic acid, mannose, and TAT peptide, which were hydrophobic, hydrophilic, and amphiphilic, respectively. The graft polymers were characterized and studied for their in vitro properties. In-vivo transfection study was further conducted on the nanomicelles. Finally, VGF polyplexed with our superior polymeric micelle was delivered through both the intranasal and intravenous routes. In this study, we compared the expression of VGF in the brain upon administration either intravenously or intranasally.

## 2. Materials and Methods

### 2.1. Materials

Chitosan (Mw = 50 kDa, 85% deacetylated) (CS) 3-(4,5-dimethylthiazol-2-yl)-2,5-diphenyl-tetrazolium bromide (MTT), and fluorescein 5-isothiocyanate (FITC), Deoxyribonuclease I from bovine pancreas, Triton X-100, 2,4,6-Trinitrobenzenesulfonic acid solution (TNBSA), was purchased from Sigma-Aldrich (St. Louis, MO, USA), Oleic acid (OA) was purchased Spectrum Chemical (New Brunswick, NJ, USA). The 1-Ethyl-3-(3′-dimethylaminopropyl) carbodiimide (EDC·HCl) was obtained from Creosalus Inc. (Louisville, KY, USA). N-Hydroxysuccinimide (NHS), Pyrene, and heparin sodium were purchased from Alfa Aesar (Haverhill, MA, USA). Phosphate buffered saline (PBS), fetal bovine serum (FBS), and Dulbecco’s modified Eagle medium (DMEM) were acquired from Corning Incorporated (Corning, NY, USA). The 4-Isothiocyanatophenyl-alpha-D-mannopyranoside was obtained from Toronto research chemicals (Toronto, ON, Canada). Cysteine terminated TAT was purchased from Anaspec Inc. (Fremont, CA, USA). Tris-acetate-ethylenediaminetetraacetic acid (TAE) was purchased from Omega Bio-Tek Inc., Norcross, GA, USA). Mouse brain endothelial (bEnd.3) cell lines were purchased from American Type Culture Collection (ATCC, Rockville, MD, USA). Plasmid DNA encoding green fluorescent protein (gWiz-GFP) and nerve growth factor-inducible (pVGF) were acquired from Aldevron LLC (Fargo, ND, USA). VGF enzyme assay kit with reporter lysis buffer was procured from Promega (Madison, WI, USA). Micro BCA protein assay kit was bought from Pierce Biotechnology Inc. (Rockford, IL, USA). Agarose was purchased from MP Biomedicals Inc. (Aurora, OH, USA). Safe-Green™ was purchased from Applied Biological Materials (Richmond, BC, Canada). All other reagents were of analytical grade and were used without further modification.

### 2.2. Synthesis and Characterization of OA-g-CS-Man-Tat Polymer

Chitosan was grafted with oleic acid (OA) using previously reported method [23]. The OA conjugation on chitosan backbone was facilitated by carbodiimide-mediated coupling, and the carboxylic group of OA and amine of chitosan were the acting functionalities. Briefly, chitosan (0.5 gm) was completely solubilized in 0.1% acetic acid (50 mL). OA (0.3 mole/mole of glucosamine units of chitosan) was dissolved in ethanol (10 mL), EDC.HCl (5 mol/mol of OA) was used to activate OA’s carboxylic functionality, followed by the addition of NHS (5 mol/mol of OA). Activated OA was added dropwise to the chitosan solution and allowed to react for 12 h at 90 °C. At the end of the reaction, the OA grafted chitosan (OA-g-CS) conjugate was purified through dialysis membrane (MWCO: 3.5 K) for 48 h against de-ionized water. The dialyzed product was freeze-dried, washed with ethanol to remove unreacted OA, and the residual ethanol evaporated using vacuum evaporator.

Further, OA-g-CS was tagged with mannose via a phenylisothiocyanate bridge. Briefly, OA-g-CS was dispersed in deionized water. The 4-Isothiocyanatophenyl-alpha-D-mannopyranoside was added to the reaction mixture and allowed to stir for 4 h at room temperature. The reaction product (OA-g-CS-Man) was purified by dialysis in a 3.5 K membrane.

Finally, TAT peptide conjugation was achieved through a maleimide–thiol reaction. Briefly, NHS terminated Peg-Maleimide (Biochempeg, Watertown, MA, USA) was conjugated to either OA-g-CS or OA-g-CS-Man. The products (OA-g-CS-Peg-Mal or OA-g-CS-Man-Peg-Mal) were reacted with a cysteine terminated TAT to produce OA-g-CS-Tat or OA-g-CS-Man-Tat). Unreacted TAT was removed by dialysis using a 3.5 K dialysis membrane. Proton nuclear magnetic resonance (^1^H-NMR) and Fourier transform infra-red (FT-IR) spectroscopic analysis were used to confirm the formation of all desired products. For physical characterization and NMR spectroscopic analysis, samples were prepared in de-ionized water (1 mg/mL) and deuterated solvents, respectively.

### 2.3. Percentage OA Grafting on Chitosan

Percentage grafting of OA on chitosan skeleton was determined by the TNBSA assay [33]. Briefly, OA-g-CS was dissolved in DI water (0.2 mg/mL), TNBSA was added (0.02% *w*/*v*, 500 µL) and incubated for 2 h at 37 °C. The reaction was stopped by the addition of 125 µL HCl (10N). Samples were analyzed in a spectrophotometer (SpectraMax M5 microplate reader Molecular Devices, San Jose, CA, USA) at 335 nm. Chitosan was used as a control; grafting was calculated with the formula:

%GR = (1 − A_test_/A_ctrl_) × 100

where A_test_ is absorbance of OA-g-CS conjugates and A_ctrl_ is the absorbance of unmodified chitosan.

### 2.4. Critical Micelle Concentration (CMC)

Pyrene (as a hydrophobic probe) method was used for the determination of critical micelle concentration as documented earlier [23]. Briefly, Pyrene 10 µL of a 24 µg/mL pyrene solution in acetone was transferred into a clean 10 mL glass test tube and the acetone was evaporated. CS-modified polymers OA-g-CS, OA-g-CS-MAN, OA-g-CS-Tat, and OA-g-CS-Man-Tat at increasing concentrations were prepared in a 2 mL volume and added to the dried pyrene to achieve a final pyrene concentration of 0.6 µM. Each polymer–pyrene solution was sonicated for 5 min and kept away from light for 24 h. The fluorescence emission spectrum of pyrene in each sample was measured in SpectraMax M5 microplate reader (Molecular Devices, San Jose, CA, USA) at 360–460 nm and 336 nm excitation wavelength. The CMC was calculated by plotting a graph between I_373_/I_393_ and log10 concentrations of the conjugates.

### 2.5. Polymer–pDNA Polyplex Preparation and Characterization

Prepared polyplexes were subjected to physical characterization. The samples were prepared in deionized water (acidified with acetic acid to a pH of 5.5) at a concentration of 1 mg/mL. The polymer solutions were extruded through a 0.2 μm Whatman Nucleopore polycarbonate membrane (GE Healthcare, Chicago, IL, USA) held in an Avanti Mini Extruder (Avanti Polar Lipids, Inc., Alabaster, AL, USA). The average hydrodynamic diameter, zeta potential, and polydispersity index of all micelles were determined using a Zetasizer Nano ZR (Malvern Instruments, Malvern, UK).

### 2.6. N/P Ratio Optimization

The polymer–pVGF association was optimized by agarose gel retardation assay using OA-g-CS-Man-Tat polymer. Briefly, different weight ratios of polymers were polyplexed with 1 µg of pVGF. The OA-g-CS-Man-Tat–pVGF polyplex was then mixed with Safe-Green™, loaded onto a 0.8% (*w*/*v*) agarose gel, and run with 0.5× Tris-acetate-ethylenediaminetetraacetic acid (TAE) running buffer for 80 min at 80 V. At the end of the run, the gel was analyzed in a transillluminator. The lowest N/P ratio that allowed polyplex formation while sufficiently protecting DNA from DNase degradation was selected as the optimum N/P ratio.

### 2.7. DNA Binding and DNase Protection Efficiency of Different Chitosan Polymers

Briefly, Cs and all CS-modified polymers were dissolved in acidified deionized water (1 mg/mL), pVGF (1 µg) at the optimized N/P ratios. The polyplex complexes were incubated for 45 min at 37 °C and Safe-Green™ was added to polyplex samples (5:1). The stained polyplex were electrophoresed in 0.8% agarose gel at 80 V in 0.5× Tris-acetate-ethylenediaminetetraacetic acid (TAE) running buffer for 80 min.

DNase protection of pVGF in polymeric micellar formulation from DNase I was evaluated as reported earlier [24]. Briefly, 1 µg pDNA polyplexes with polymeric micelles were incubated with 1 unit of DNase I enzyme in DNase reaction buffer at 37 °C for 1 h. Post-incubation, EDTA (5 µL) was added to chelate all divalent ions present and inactivate the DNase. This was followed by incubation with heparin (20 µL, 5 mg/mL) for 2 h to release pDNA from the nanomicelle formulation. Naked pDNA was used as positive control. Stability of released pDNA was analyzed using 0.8% (*w*/*v*) agarose gel electrophoresis (80 V, 80 min).

### 2.8. Blood Compatibility Study

Ex-vivo erythrocyte compatibility of the optimized polymeric micelles was evaluated using the previously reported method [25]. Briefly, erythrocytes were isolated from freshly withdrawn blood (Sprague Dawley rats) by centrifugation (277× *g* for 10 min) followed by washing with PBS (pH 7.4) and CaCl_2_ solution (10 mM). Erythrocytes (2% *v*/*v*) were incubated with different concentrations (3–1000 μg/mL) of polymeric micelles for 1 h at 37 °C. Post-incubation, the cell suspension was centrifuged (277× *g*, 10 min), the supernatant was analyzed to quantify released hemoglobin using a SpectraMax M5 microplate reader (Molecular Devices, San Jose, CA, USA) at 540 nm. Triton X-100 and PBS were used as a positive and negative control, respectively. Relative hemolysis was calculated by considering Triton X-100 as 100% hemolysis.

### 2.9. In-Vitro Cytotoxicity

Cellular toxicity of CS, OA-g-CS, OA-g-CS-Man, OA-g-CS-Tat, and OA-g-CS-Man-Tat was evaluated against bEnd.3 cells by following a previously reported method [26]. Briefly, bEnd.3 cells (1 × 10^4^/well) were seeded in 96 well plates and incubated for 24 h prior to the study. Cells were treated with different micellar concentrations (125, 250, 500, and 1000 μg/mL) in DMEM culture media. After four hours the DMEM was replenished with DMEM culture medium containing 10% Fetal bovine serum. Further, cells were incubated for 48 h, and 10 µl of MTT (5 mg/mL) was added to evaluate the cell viability. After 3 h of incubation, MTT solution was removed and DMSO was added to dissolve the formazan crystals. Samples were analyzed at 570 nm in a microplate reader. Untreated cells were used as the control for the study.

Cell viability = (A-treated/A-untreated) × 100

where A-treated is the average absorbance of wells incubated with polymer and A-untreated is the average absorbance of the control wells.

### 2.10. In Vitro Transfection

Transfection efficiency of polymeric micelles were evaluated against primary astrocytes and neurons (cultured from 1-day old rat pups) and bEnd.3 cells separately. Approximately 1 × 10^6^ cells were seeded per well in 6 well plates 24 h prior to the experiment. Cells were treated with naked pVGF–pGFP alone or pVGF–pGFP polyplexed with our polymers. Cells were incubated for 6 h after which the transfection media was replaced with DMEM culture medium containing 10% Fetal bovine serum and further incubated for 48 h. For pGFP transfection, cells were visualized under a Leica DFC 3000G fluorescence microscope (Leica biosystems, Wetzlar, Germany). Plasmid VGF transfection was quantitatively analyzed using ELISA Kit (MyBiosource, San Francisco, CA, USA).

### 2.11. In Vivo Studies

#### 2.11.1. Animals

C57Bl/6 J mice were used for the in-vivo studies, protocol (#A21015) was approved by the institutional animal care and use committee, North Dakota State University, Fargo, ND, USA. Following NIH and Institutional Animal Care and Use Committee (IACUC) requirements, the animals were housed in the animal care facility. The animal room was maintained at a controlled temperature (22 ± 2 °CF) with 12-h light and 12-h darkness cycles. The mice had free access to standard pelleted food and water through the study.

#### 2.11.2. Comparative In-Vivo VGF Transfection and Biocompatibility

Intranasal administration procedure was followed as reported earlier [34,35] with slight modifications. Briefly, 4–8 weeks old male and female C57Bl/6 J mice were placed in eight groups (*n* = 6). Of these eight, three groups (group number 1, 2, and 3) were treated with nanomicelle formulations (CS–pVGF, OA-g-CS–pVGF, and OA-g-CS-Man-Tat–pVGF, respectively), through the nasal route. The other three groups (group numbers 4, 5, and 6) received similar formulations through the intravenous route. Both control groups were treated with saline through both routes.

The three micelle formulations selected for this study (CS–pVGF, OA-g- CS–pVGF, and OA-g-CS-Man-Tat–pVGF) were administered through intranasal and intravenous routes. Saline-treated group was used as the control. Polyplex formulations were administered in 3 μL volumes into alternate nostrils at 30 s intervals in an anesthetized mouse. Mice were sacrificed 5 days after treatment, and highly perfused body organs were carefully harvested, including brain, kidney, spleen, lungs, liver, and heart. The brain was rinsed and homogenized, followed by centrifugation at 200× *g* for 10 min. The supernatant was used to analyze VGF expression using ELISA Kit (MyBiosource, San Francisco, CA, USA).

The harvested organs in the study were rinsed with PBS, fixed in 10% buffered formalin, embedded in paraffin, and used for histopathological analyses with hematoxylin and eosin staining.

### 2.12. Statistics

All data are denoted as mean ± standard deviation (SD). All statistical analysis was performed using Student pair *t* test or Tukey’s test following one-way ANOVA. *p*-values below 0.05 were considered statistically significant.

## 3. Results

### 3.1. Synthesis and Characterization of OA-g-CS-Man-Tat Polymer

**Figure 1:**
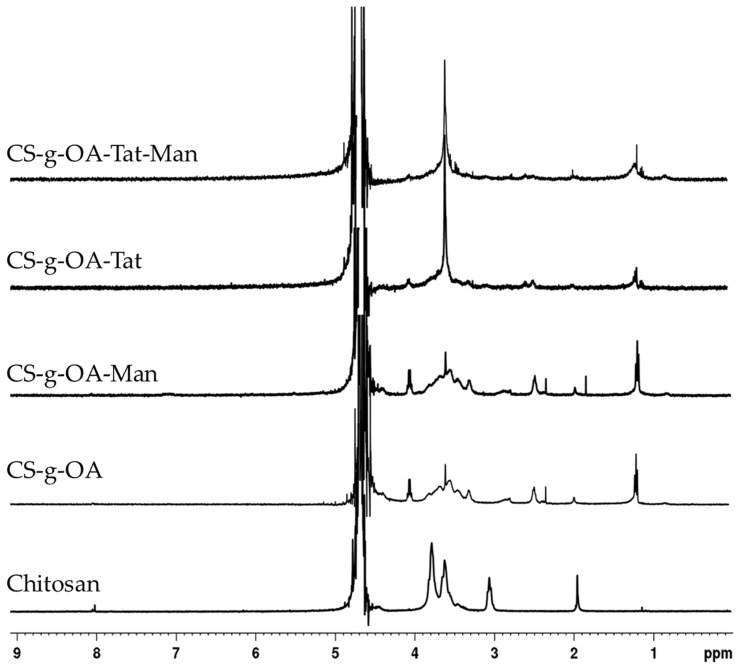
The ^1^H-NMR spectra of CS and synthesized OA-g-CS, OA-g-CS-Man, OA-g-CS-Tat, and OA-g-CS-Man-Tat conjugates.

**Figure 2:**
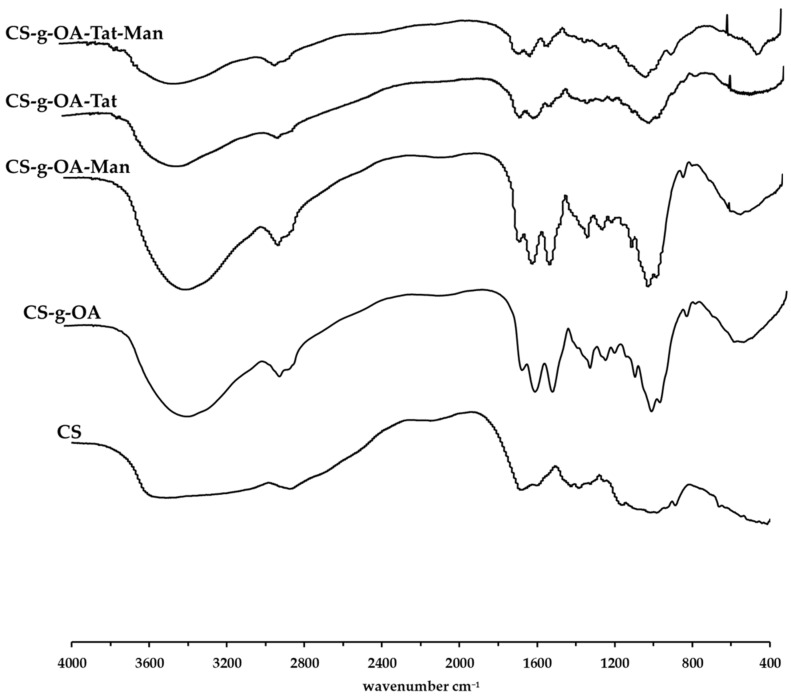
FT-IR spectra of CS and synthesized OA-g-CS, OA-g-CS-Man, OA-g-CS-Tat, and OA-g-CS-Man-Tat conjugates.

The OA-g-CS-Man-Tat conjugates were synthesized sequentially; first, OA was conjugated on the CS backbone. The underlying mechanism was an amide bond formation between carboxylic functionality of OA and amine of CS, facilitated by EDC/NHS mediated coupling reaction. Further, amine functionalities of CS were tagged with mannose followed by Tat peptide conjugation. Both ^1^H NMR and FT-IR spectroscopy were used to confirm the conjugation reactions (Figure 1 and Figure 2). All samples for ^1^H NMR were dissolved in 1% deuterium chloride in Deuterium oxide (D_2_O) conducted on a 400 MHz Bruker scientific instrument (Bruker Scientific, Billerica, MA, USA).

The ring protons (H-3, 4, 5, 6, 6′) of chitosan were observed to resonate at 3.4−4.0 ppm, as depicted in the spectra (Figure 1). The characteristic chemical shifts of OA in OA-g-CS conjugate were at 1.2 ppm, and methylene protons adjacent to amide bond at 2.5 ppm. Mannose conjugation was confirmed by the appearance of protons at 1.8 ppm. Integrated spectra are provided as supplementary data (Supplementary Materials Figures S1–S5).

Further, FT-IR was used to assess the synthesized products. Absorption peaks of CS at around 3511 cm^−1^ is attributed to the N-H stretching of primary amines present in chitosan. The absorption peaks present at around 2873 cm^−1^ are attributed to the -C-H- stretching whilst the absorption peak at around 3511 cm^−1^ is attributed to the primary alcohol present on the glucosamine ring of chitosan. Upon conjugation, the prominent peaks that appear at ~1653 cm^−1^ are associated to the amide -C=O stretch that is formed upon conjugation as well as the -C=C- double bond present in the oleic acid group. Primary alcohol -C-O stretch absorption was observed at ~1070 cm^−1^. Tat conjugation resulted in a peak at 3700 cm^−1^.

### 3.2. Percentage OA Grafting on Chitosan

The degree of fatty acid grafting on chitosan was determined using the TNBSA bio-reagent. TNBSA reacts with primary amine groups present in chitosan to form a highly chromogenic product. The absorbance of this derivative can be measured using absorption spectroscopy and the percentage amine group replacement can be calculated. The grafting percentage of oleic acid on chitosan was found to be 21 ± 0.069%

### 3.3. Critical Micelle Concentration

The critical micelle concentration is an important parameter, which defines the self-aggregating properties of polymers. Polymeric micelle formation involves self-assembling of amphiphilic block polymers. Chitosan is a hydrophilic polymer, but surface modification with fatty acids can transform it into an amphiphilic block polymer, which can reassemble in aqueous solution. The critical micelle concentrations for OA-g-CS, OA-g-CS-Man, OA-g-CS-Tat, and OA-g-CS-Man-Tat conjugates were found to be 97.2, 125, 79.9, and 83.3 (μg/mL), respectively, as seen from Table 1.

### 3.4. Polymer–pDNA Preparation and Characterization

Size and zeta potential of polymeric micelles define the gene delivering efficacy; cationic charge of the polymeric micelles is desirable for polyplex formation and interaction with the cell membrane [19]. As can be seen in Table 2 and Supplementary Materials Figures S6–S11, functionalization of chitosan did not drastically impact the hydrodynamic diameter. All polymers formed micelles of size approximately 200 nm. (Table 2).

### 3.5. N/P Ratio Optimization

**Figure 3:**
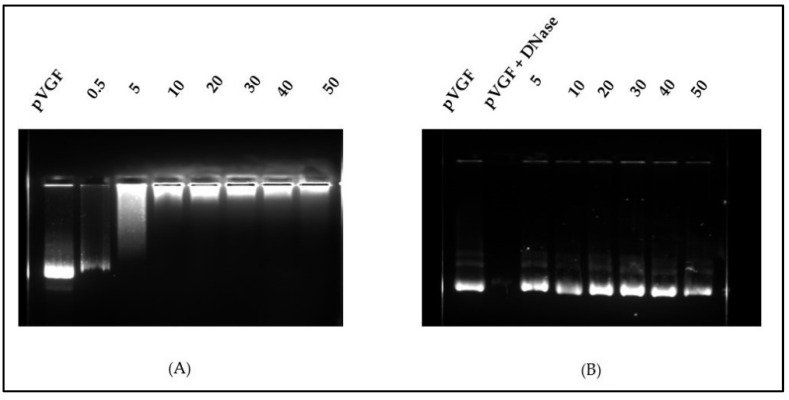
(**A**) Analysis of OA-g-CS-Man-Tat–pVGF complex formation at different weight ratios by agarose gel electrophoresis. (**B**) DNase I protection assay showing protection of DNA from degradation by polymers at the different N/P ratios.

The N/P ratio is an important parameter that informs on the extent of association and ability to release free pDNA. The suitable N/P ration was accessed using our final formulation OA-g-CS-Man-Tat polyplexed to pVGF and with naked pVGF as a positive control. As shown from Figure 3A, an N/P ratio above 10 was optimum for polyplex formation between the plasmid and polymer. DNase activity was further used to ascertain the protective effects of the polymer at the different ratios, and all concentrations showed good ability to protect pVGF from digestion by DNase, as seen in Figure 3B.

### 3.6. DNA Binding and DNase Protection Efficiency of Different Chitosan Polymers

**Figure 4:**
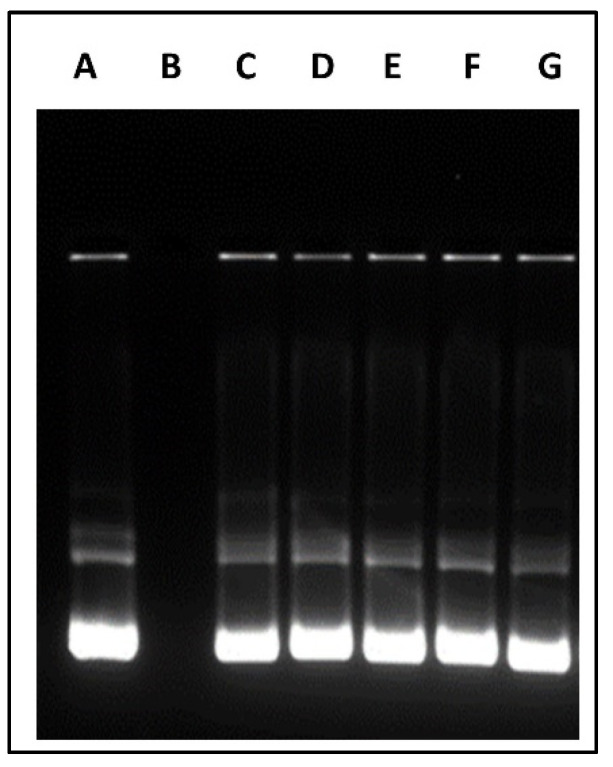
DNase protecting effect of different polymeric micelles containing pVGF. Column (**A**) Untreated pVGF, column (**B**) pVGF treated with DNase, column (**C**) CS–pVGF column (**D**) OA-g-CS–pVGF, column (**E**) OA-g-CS-Man–pVGF, column (**F**) OA-g-CS-Tat–pVGF, Column (**G**) OA-g-CS-Man-Tat–pVGF.

The formation of stable polyplex between the modified polymers and pVGF was examined by agarose gel electrophoresis. Aside from transport, a gene delivery nanocarrier system should protect the gene from enzymatic degradation and deliver it to the desired site. We assessed the ability of all our polymers to polyplex and protect pDNA at the N/P ratio of 10:1. As shown in Figure 4, naked pVGF was vulnerable against DNase, while multifunctional polymeric micelles protected the pVGF from DNase digestion.

### 3.7. Blood Compatibility Study

**Figure 5:**
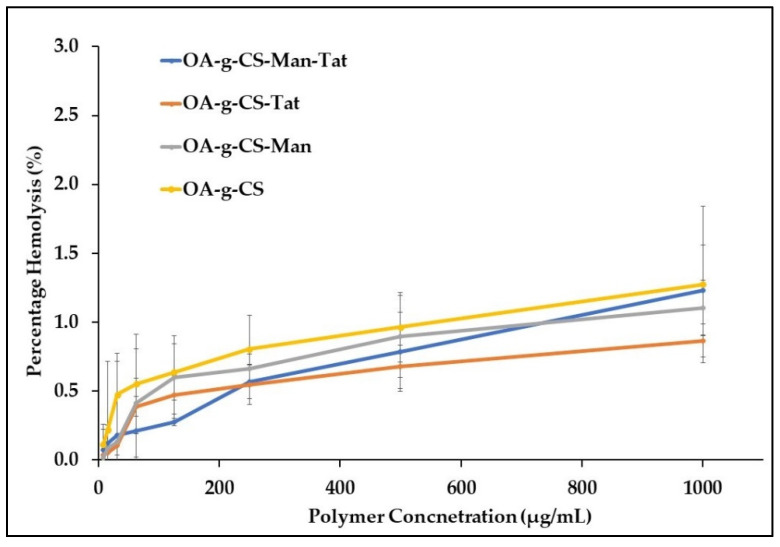
Hemolytic activity of chitosan-modified polymers at different concentrations. A total of 1% (*v*/*v*) Triton X-100 was used as a positive control (hemolytic activity was set as 100%) at 37 °C.

**Figure 6:**
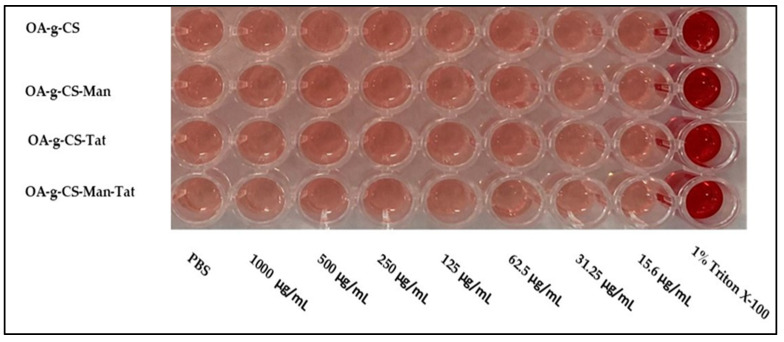
Images of hemolysis caused following 1 h incubation with different polymers at different concentrations.

It was necessary to evaluate the blood compatibility of the synthesized polymers. The hemolysis, caused by OA-g-CS, OA-g-CS-Man, OA-g-CS-Tat, and OA-g-CS-Man-Tat, was determined by measuring the hemoglobin released by erythrocytes upon exposure to the different formulations. As seen in Figure 5, hemolytic activity remained below 2%, which suggests that polymers did not harm the erythrocytes. Moreover, Figure 6 visually corroborates the observed outcomes in Figure 5; erythrocytes treated with PBS were similar in appearance to cells treated with formulations at different concentrations.

### 3.8. In Vitro Cytotoxicity

**Figure 7:**
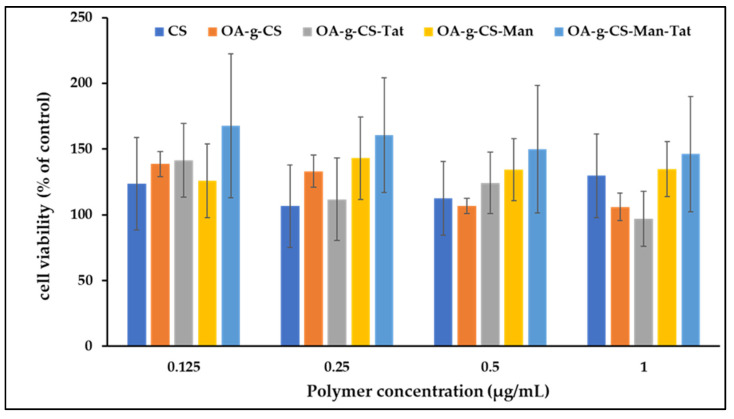
In vitro cytocompatibility of CS, OA-g-CS, OA-g-CS-Man, OA-g-CS-Tat, and OA-g-CS-Man-Tat polymeric micelles at different concentrations (0.125–1000 µg/mL) in bEnd.3 cells. MTT was used to evaluate cell viability, data presented as mean ± SD (n = 4).

Polymer formulations did not exert any noticeable toxicity on the bEnd.3 cells (Figure 7). At all concentrations of the different polymers used (125–1000 µg/mL), the cell remained over 95% viable.

### 3.9. In Vitro pGFP and pVGF Transfection

**Figure 8:**
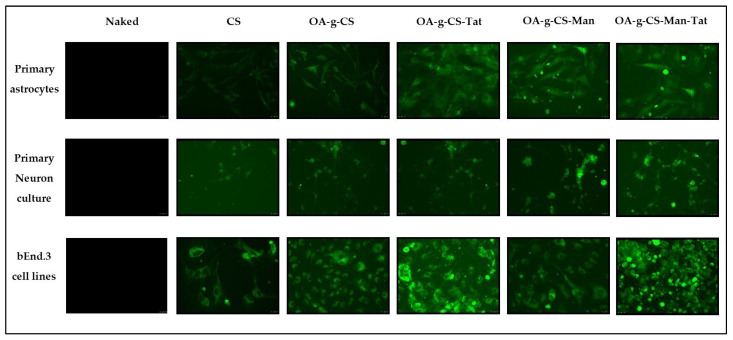
Qualitative analysis of in vitro transfection efficiency of naked pGFP, CS–pGFP, OA-g-CS–pGFP, OA-g-CS-Man–pGFP, OA-g-CS-Tat–pGFP, and OA-g-CS-Man-Tat–pGFP against primary astrocytes, primary neurons, and bEnd.3 cells. Images were taken at 20× magnification.

Primary astrocytes, primary neurons, and bEnd.3 cells were used to demonstrate and evaluate the in vitro transfection efficacy of multi-functionalized polymeric micelles. All cell types were treated with naked pGFP, CS–pGFP, OA-g-CS–pGFP, OA-g-CS-Man–pGFP, OA-g-CS-Tat–pGFP, and OA-g-CS-Man-Tat–pGFP polyplexes in order to aid qualitatively accessing transfection. Qualitative evidence of pGFP using different formulations is seen in Figure 8.

**Figure 9:**
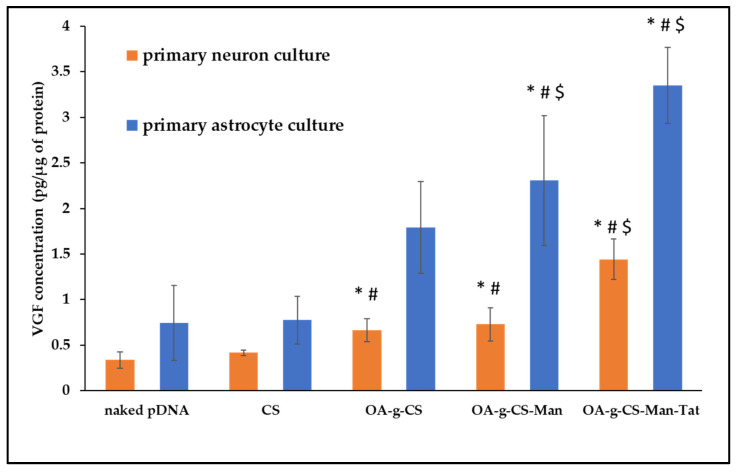
VGF expression levels in primary astrocytes and primary neurons after treatment with CS–pVGF, OA-g-CS–pVGF, and OA-g-CS-Man-Tat–pVGF polyplexes. Results presented as mean ± SD (*n* = 4). (“*” indicates significantly (*p* < 0.05) different from naked pDNA “#” indicates significantly (*p* < 0.01) different from respective naked pDNA and “$” indicates significantly (*p* < 0.001) different from respective naked pDNA).

To measure transfection efficiency quantitatively, pVGF was polyplexed with formulations CS, OA-g-CS, OA-g-CS-Man, and OA-g-CS-Man-Tat. Following a 48-h incubation period, ELISA was performed to analyze the relative expression of VGF post-normalization with the total protein content of the cells. The CS–pVGF polyplex was a minor transfecting complex against all three types of cells, while OA-g-CS-Man-Tat–pVGF was the most transfecting polyplex (Figure 9).

### 3.10. In-Vivo VGF Transfection

**Figure 10:**
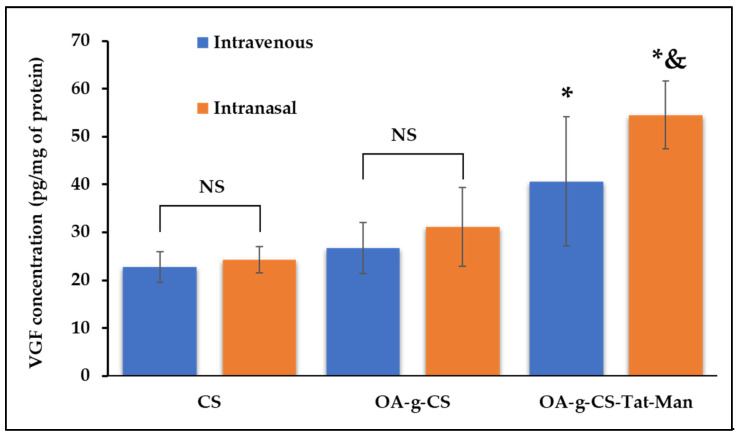
In-vivo VGF expression after treating C75BI/6 J mice with CS–pVGF, OA-g-CS–pVGF, and OA-g-CS-Man-Tat–pVGF polyplexes using the intranasal and intravenous route. Results are presented as mean ± SD (*n* = 6). (“*” indicates significantly (*p* < 0.05) different from intravenous CS “&” indicates significantly (*p* < 0.05) different from intravenous OA-g-CS-Man-Tat–pVGF). NS represents non significance.

Equal numbers of both male and female C75BI/6 J mice were selected for the study, because the intranasal route is well developed in both genders. Organs of treated animals were harvested 5 days after administration of similar doses of the gene. VGF expression was analyzed using VGF ELISA kit (Promega, Madison, WI, USA). As seen in Figure 10, significantly (*p* < 0.05) higher transfection was observed in mice treated intranasally using OA-g-CS-Man-Tat–pVGF.

**Figure 11:**
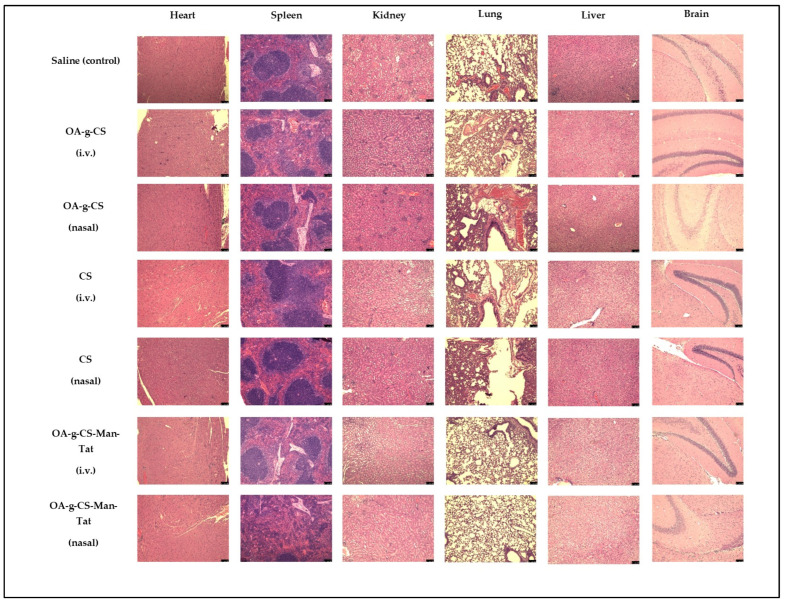
H and E staining of heart, spleen, kidney, lung, liver, and brain, showing no toxicity from different groups after drug administration. Images were taken at 20× magnification and the scale bar is 75 µm.

Highly perfused organs were harvested and stained for analysis. When compared to the control groups (saline-treated groups), no toxicity was observed, and cell morphology remained intact and did not show any signs of damage or harm (Figure 11).

## 4. Discussion

Surface modification of nanoparticles with ligands is widely exploited to target a variety of disorders [36,37] Targeting the brain for treatment of neurological disorders is challenging through the common routes of administration (i.v. and oral) [38,39]. This is owing to the tight structural integrity of the BBB’s architectural cells (astrocytes, pericytes, and endothelia). There is an abundant amount of literature that indicates that administration through the intranasal route can bypass the BBB, due to the olfactory and trigeminal nerves, which connect the olfactory epithelium in the nasal cavity to the brain [40]. In addition to this, the intranasal route is known to be safe and non-invasive compared to other approaches to target the brain such as intracranial injection [41]. Further, most of the pharmaceutical bioactive agents (due to their pharmacokinetic properties) cannot cross the blood barrier by themselves, causing the unmet need for brain targeted delivery systems. Currently, the only approved protein or ligand for brain delivery is the adeno associated virus-based gene therapy for infantile spinal muscular atrophy with single i.v. administration [42]. The success achieved so far marks a path for the development of more effective delivery systems with high efficiency for delivering neurotherapeutics (both drugs and gene) to the brain. Ligand–receptor-based approaches have been explored extensively for effective drug and gene delivery to the brain through BBB [15,43].

Mannose-based nanocarriers are among the most studied drug delivery platforms for active brain targeting because of the abundance of GLUT-1 receptors on cells in the BBB [26,44]. Similarly, HIV 1-Tat is a regulatory protein that activates different receptors on endothelial cells [45]. Therefore, it can be used as a targeting ligand for active targeting to the brain. Further, mannose receptor (GLUT-1) and Tat receptors (TAR) are present in neurons to improve drug uptake in the brain via olfactory and trigeminal nerves. Thus, both routes of administration, i.v. and intranasal, are likely to encounter the same targeting receptors.

The synthesized polymers freely formed micelles with cationic charge and size approximately 200 nm, which makes these micelles efficient for gene delivery [23]. Nanoparticles having size <200 nm have shown better cellular uptake [46]. Although decreased by surface modification, the zeta potential of all formulations remained positive. This is essential to aid cell internalization [47] and pDNA association [48]. The polydispersity index of the multifunctional OA-g-CS-Man-Tat polymeric micelles was found to be less than 0.3. This demonstrates stability, less aggregation, and mono-dispersity in solvent systems [46]. Synergistically, nanometric size and cationic zeta potential increase the cellular uptake through the cellular membrane. The change in the zeta potential of conjugates is mainly due to exhaustion of the free primary amine groups previously present on the unmodified chitosan backbone.

The critical micelle concentration of a polymer depends on its chemical characteristics, molecular weight, and ratio of hydrophilic-to-hydrophobic segments [49]. The ability to form micelles is also a crucial characteristic of polymeric drug delivery systems. Thus, we investigated the critical micelle concentration of our formulations at room temperature. As seen from Table 1 and Supplementary Materials Figure S12, the formation of micelles was observed in all our formulations at reasonably lower concentrations when investigated with pyrene as a hydrophobic probe. This orientation of polymers provides stealth covering to the bio-actives, which protects them from the enzymatic degradation in the biological milieu [50]. An increase in hydrophilic properties upon conjugation with mannose drastically increased the critical micelle concentration. Similarly, conjugation with HIV 1-Tat peptide lowered the critical micelle concentration, due to the increase in hydrophobicity upon HIV 1-Tat conjugation. However, conjugation with both mannose and HIV 1-Tat lowered the critical micelle concentration due to the presence of more hydrophobic characters (from the HIV1-Tat) than hydrophilic character [51].

The N/P ratio of chitosan to plasmid VGF was determined to identify the optimum ratio of polymer to plasmid that was enough to protect and release the VGF into intracellular space. The N/P ratio of 10 was chosen for OA-g-CS-Man-Tat. The N/P ratio is crucial because although a tight complex is desirable for transport and protection, it should also be sufficient to ensure the release of the plasmid at the desired site [19,20]. This N/P ratio was used to access the DNA binding and DNase protection ability of all other formulations. A good drug carrier should not only deliver the drug to the target site but also deliver it in a wholesome manner. We investigated our polymeric formulations’ ability to protect the plasmid DNA from degradation in the presence of DNase. Although conjugation with Mannose, Tat, and oleic acid did decrease the zeta potential, it did not greatly affect the polyplex formation and cargo protection at the selected N/P ratio, as seen in Figure 4. All prepared polymeric micelles efficiently protected VGF from enzymatic degradation by DNase.

Erythrocyte lysis takes place due to an interaction between cationic charged polymeric micelles and negatively charged membrane. Possible nonspecific interactions of positively charged polymers and blood components severely limits their use in in-vivo settings. Chitosan possesses a high cationic charge, but the surface modification restricts its direct exposure to erythrocytes. From our study, we observed less than 2% hemotoxicity when our synthesized polymers were exposed to erythrocytes. According to the ISO/TR 7406, 5% hemotoxicity caused by biomaterials falls within the critical safe limit, making OA-g-CS-Man-Tat polymeric micelles safe for further use [52].

Every drug delivery tool should be safe. Chitosan has been used chiefly due its superior properties of being biocompatible, biodegradable, and nontoxic at the doses used in drug delivery. We confirmed that our conjugation did not impact this safety profile. When tested against bEnd.3 cell lines, higher cell confluences were observed, (Figure 7) indicating that our formulation did not adversely affect the viability of the cells. The possible explanation for non-toxicity is the surface modification of the Chitosan backbone with Oleic acid, Mannose, and Tat. Further, previous studies reported that chitosan and its modified polymers can improve cell viability [16,19,23]. Our findings are in line with previous findings and demonstrate the nontoxic nature of our synthesized polymers.

Our primary goal was to transfect astrocytes and neurons in-vivo. Thus, we had to mimic this transfection in vitro. To allow visualization of our transfection, green fluorescent protein (GFP) was used as a model gene to perform the transfection studies. As seen in Figure 8, when introduced to primary astrocyte, neurons, and bEnd.3 cell (observed as low fluorescence), there was reasonable uptake of all formulations. Higher fluorescence was observed for OA-g-CS-Tat in bEnd.3 compared to all other formulations in similar cells. The quantitative in vitro study was performed with VGF. Because VGF is a secreted protein, we assessed the concentrations that were both present in the media of the cultured cells and normalized these values to the total protein expressed by the cells. In vitro quantitative uptake studies were only performed in primary astrocytes and neurons. Higher transfection levels were achieved in astrocytes compared to neurons. The receptor-ligand-based mechanism is the potential driving force for the polyplex to transfect the cells. The VGF expression through them was considerably lower than that of the OA-g-CS-Man-Tat–pVGF in both neurons and astrocytes. The OA-g-CS-Man-Tat–pVGF polyplex-treated neurons had ~2.5 times higher VGF expression than the OA-g-CS–pVGF polyplex. This is due to the higher site-specific delivery of the polyplex. The multifunctional modification provides multiple ligands Mannose and Tat to interact with targeting receptors. This ligand–receptor interaction leads to higher transfection of VGF to a higher extent than the OA-g-CS–pVGF polyplex.

Transport of functionalized nanoparticles into the brain is essential to ensure sufficient expression of the transported gene. The route in which a drug is administered can affect its biodistribution and more significantly the brain uptake. Due to the high transfection levels achieved in the in vitro studies, a comparative in-vivo study was performed to evaluate the transfection potential of the multifunctional OA-g-CS-Man-Tat–pVGF polyplex into brain.

The mice group, treated intranasally with CS–pVGF, showed slightly higher but not significantly different (*p* > 0.05) VGF expression (24.3 ± 2.7 pg/µg protein in brain) than intravenously administered CS–pVGF (22.8 ± 3.2 pg/µg protein in brain). Similarly, the mice group treated with OA-g-CS–pVGF polyplex, whether intravenously (26.7 ± 5.3 protein in brain) or intranasally (31.1 ± 8.2 pg/µg protein in brain), did not show any significant difference (*p* > 0.05) in VGF expression upon administration through the different routes. In addition, the VGF expression levels observed with OA-g-CS–pVGF polyplex treatment were not significantly different (*p* > 0.05) when compared to the CS–pVGF-treated groups, regardless of route.

On the other hand, OA-g-CS-Man-Tat–pVGF-treated mice group showed 40.6 ± 13.5 pg/µg protein in the brain when administered via i.v. route. This value was only significantly different (*p* < 0.05) from the CS–pVGF i.v. (*p* = 0.009) and intranasally (*p* = 0.014) treated groups. Compared to both the i.v. and intranasally administered OA-g-CS–pVGF, no significant difference (*p* > 0.05) was observed, although the mean VGF expression for OA-g-CS-Man-Tat–pVGF (i.v.) was higher. When administered intranasally, OA-g-CS-Man-Tat–pVGF-treated mice group produced a VGF expression of 54.5 ± 7.0 pg/µg protein in brain; this value was significantly higher (*p* < 0.05) than all other groups regardless of route of administration. The superior uptake and VGF expression in the brain displayed by our micellar formulations when administered intranasally is not only due to the anatomy of the intranasal route but also due to Mannose and Tat ligand tagging, making the micelles suitable for brain targeting via receptor–ligand interaction.

Our nanoparticles are biocompatible in vitro. However, it was important to investigate if any toxicity did occur during in-vivo administration. Throughout the study, all mice were checked for signs of distress, such as lethargy, huddled posture, difficulty breathing, and severe weight loss (more than 15% body weight). None of these signs were observed and the average weight of animals in the study remained constant at 20 ± 2 g for females and 25 ± 2 for males (data not shown). In addition, the heart, spleen, kidney, lung, liver, and brain were subjected to H and E staining to investigate for any signs of inflammation or tissue distress. As seen from Figure 11, tissues from these organs showed no signs of damage or inflammation.

Sensory neurons of airway epithelia and olfactory nerve endings are the primary target sites for drug delivery via the intranasal route [40]. It directly passes the drug into the brain through the trigeminal and sensory nerve endings. At the same time, the presence of BBB restricts the drug uptake to the brain via i.v. administration. It is well-known that pharmaceutical bio-actives absorbed by the nasal mucosa show systemic and local effects, and can also be used for brain targeting. Our in-vivo transfection results confirm the notion of improved brain delivery of genetic material over the intravenous route.

## 5. Conclusions

We synthesized and characterized the graft polymers for VGF delivery to the brain through intravenous and intranasal routes. The functionalized polymers formed micelles of ~200 nm size, positive zeta potential and PDI <0.3. This study shows evidence of non-toxicity, higher VGF binding affinity, and DNase protection ability of multi-functionalized polymeric micelles. In vitro transfection outcomes showed significantly (*p* < 0.001) higher VGF transfection with multi-functionalized polymeric micelles in primary astrocytes and neuronal cells over non-functionalized polymeric micelles. Further, intranasally administered OA-g-CS-Man-Tat–pVGF in-vivo in mice demonstrated a significantly higher (*p* < 0.05) VGF expression in the brain in comparison to intravenous administration. In conclusion, this study demonstrates the potential of multi-functionalized polymeric micelles for the targeted gene delivery to the brain through intranasal administration.

## Figures and Tables

**Table 1:** Critical micelle concentration of different polymers.

Polymer	CMC (μg/mL)
OA-g-CS	97.2
OA-g-CS-Man	125
OA-g-CS-Tat	79.9
OA-g-CS-Man-Tat	83.3

**Table 2:** Size distribution, zeta potential, and polydispersity index of the synthesized conjugates. Data shown as mean ± SD.

Polymer	Average Hydrodynamic Diameter ± SD (nm)	The Polydispersity Index (PDI) ± SD	ζ Potential ± SD (mV)
CS	214.2 ± 10.12	0.30 ± 0.056	22.4 ± 6.88
OA-g-CS	196.98 ± 15.40	0.27 ± 0.03	17.80 ± 4.68
OA-g-CS-Man	202.6 ± 5.69	0.27 ± 0.03	6.31 ± 0.606
OA-g-CS-Tat	223.64 ± 3.20	0.24 ± 0.02	15.2 ± 0.61
OA-g-CS-Man-Tat	214.35 ± 1.42	0.21 ± 0.01	6.30 ± 4.55

## Data Availability

Not applicable.

## Supplementary Materials

The following supporting information can be downloaded at: https://www.mdpi.com/article/10.3390/biomedicines10020493/s1. Figure S1: Particle size distribution for different chitosan polymer formulations using dynamic light scattering method. Figure S2: Zeta potential ddistribution for chitosan using dynamic light scattering method. Figure S3: Zeta potential distribution for Oleic acid grafted chitosan using dynamic light scatter-ing method. Figure~S4: Zeta potential distribution for OA-g-CS-Man using dynamic light scattering method. Figure S5: Zeta potential distribution for OA-g-CS-Tat using dynamic light scattering method. Figure S6: Zeta potential distribution for OA-g-CS-Man-Tat using dynamic light scattering method. Figure S7: ^1^HNMR of Chitosan. Figure S8: ^1^HNMR of OA-g-CS. Figure S9: ^1^HNMR of OA-g-CS-Man. Figure~S10: ^1^HNMR of OA-g-CS-Tat. Figure S11: ^1^HNMR of OA-g-CS-Man-Tat. Figure S12: Fluorescence spectra of hydrophobic probe pyrene with increasing concentration of CS-g-OA.
