# Supplementary material for: Synthesis and Characterization of Fatty Acid Grafted Chitosan Polymeric Micelles for Improved Gene Delivery of VGF to the Brain through Intranasal Route
Source: Biomedicines. 2022 Feb 19;10(2):493. doi: 10.3390/biomedicines10020493 (PMC8962415; doi:10.3390/biomedicines10020493)
Supplement: Supplementary file 1 [file biomedicines-10-00493-s001.zip › biomedicines-1566327-supplementary.pdf]

biomedicines

MDPI

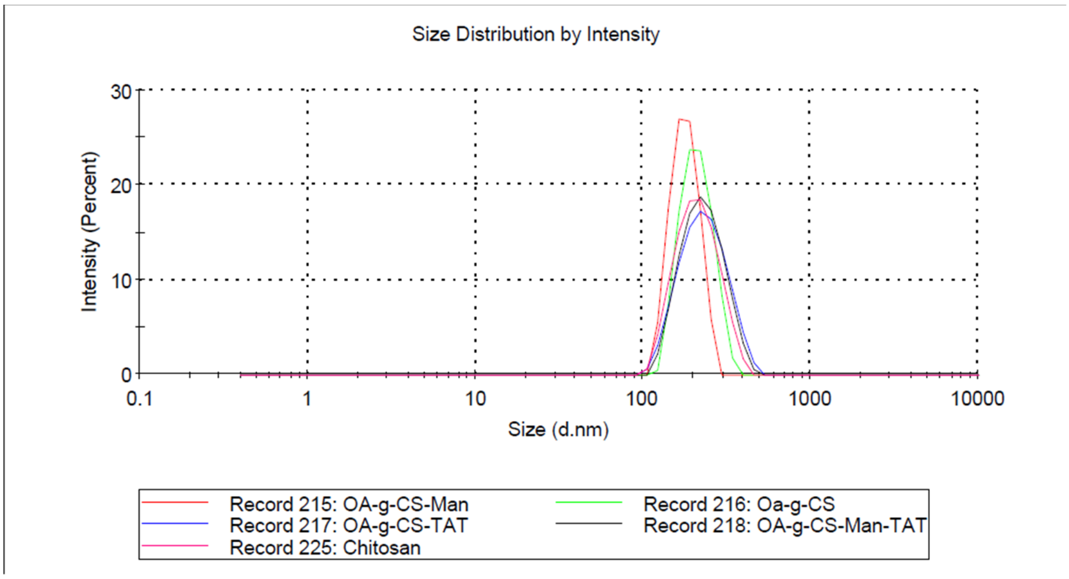

Figure S1. Particle size distribution for different chitosan polymer formulations using dynamic light scattering method.

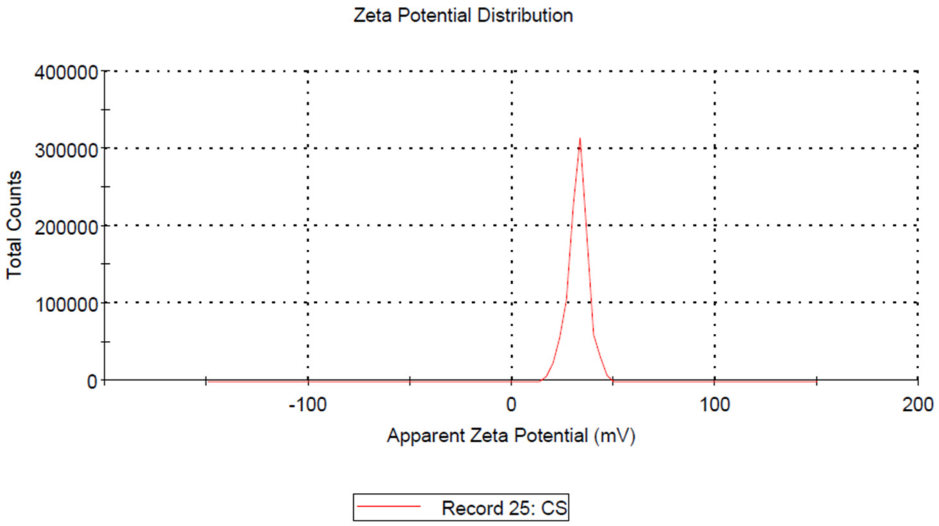

Figure S2. Zeta potential ddistribution for chitosan using dynamic light scattering method.

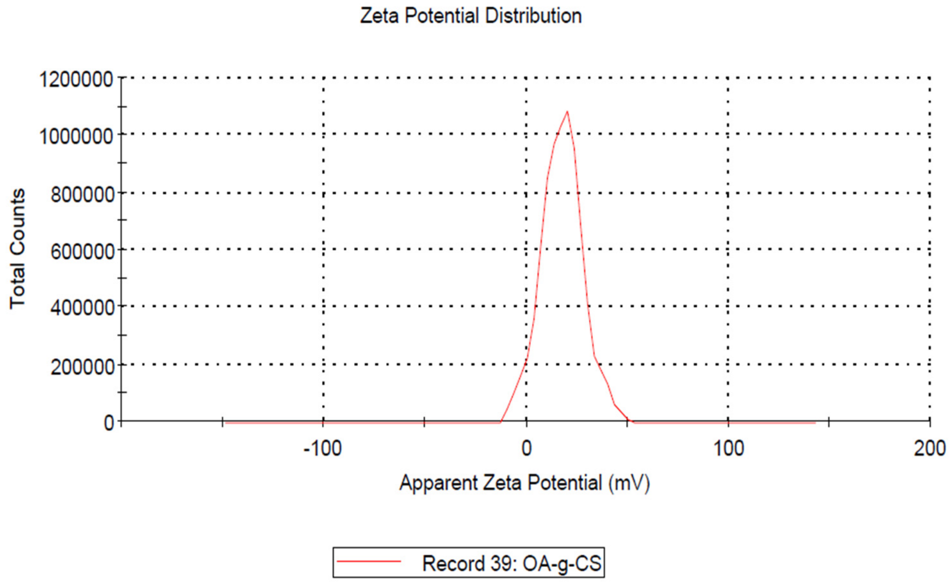

Figure S3. Zeta potential distribution for Oleic acid grafted chitosan using dynamic light scattering method.

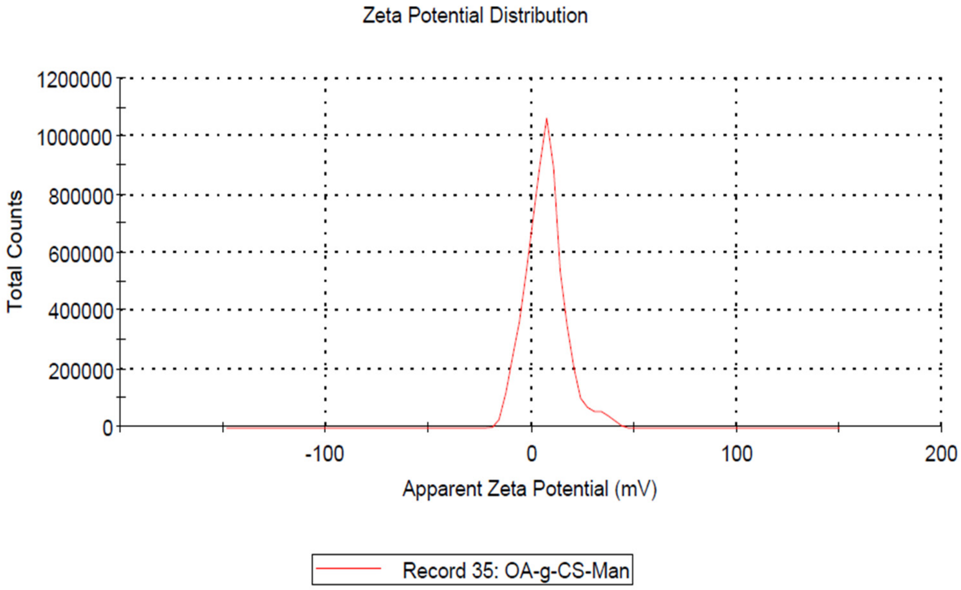

Figure S4. Zeta potential distribution for OA-g-CS-Man using dynamic light scattering method.

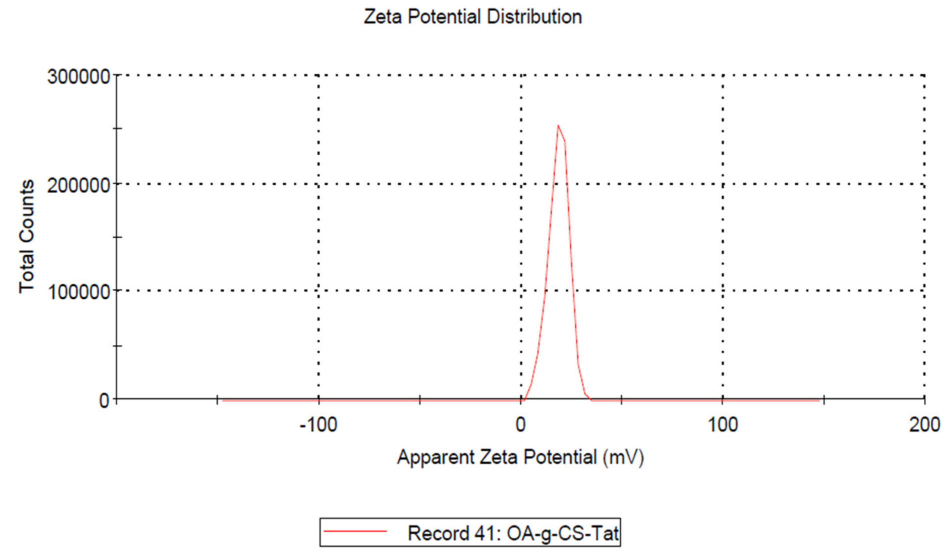

Figure S5. Zeta potential distribution for OA-g-CS-Tat using dynamic light scattering method.

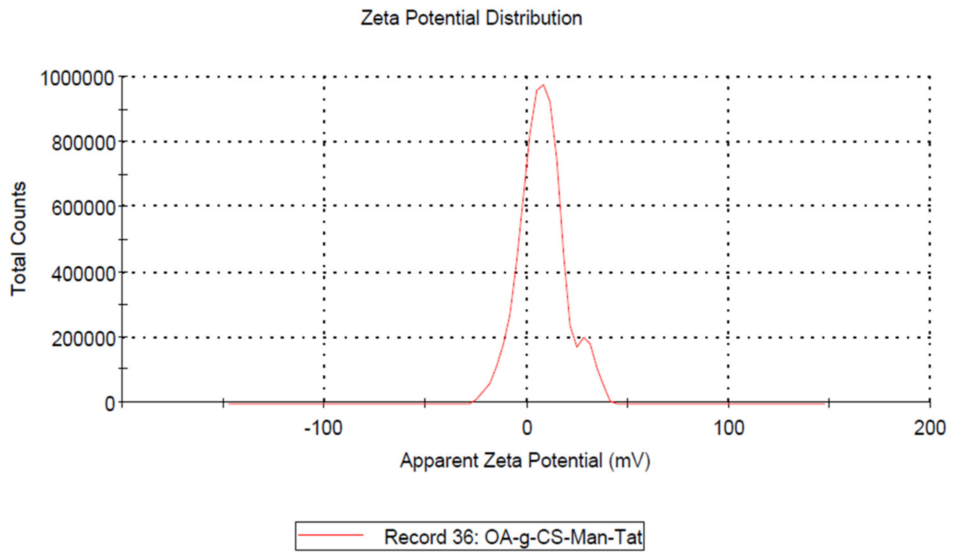

Figure S6. Zeta potential distribution for OA-g-CS-Man-Tat using dynamic light scattering method.

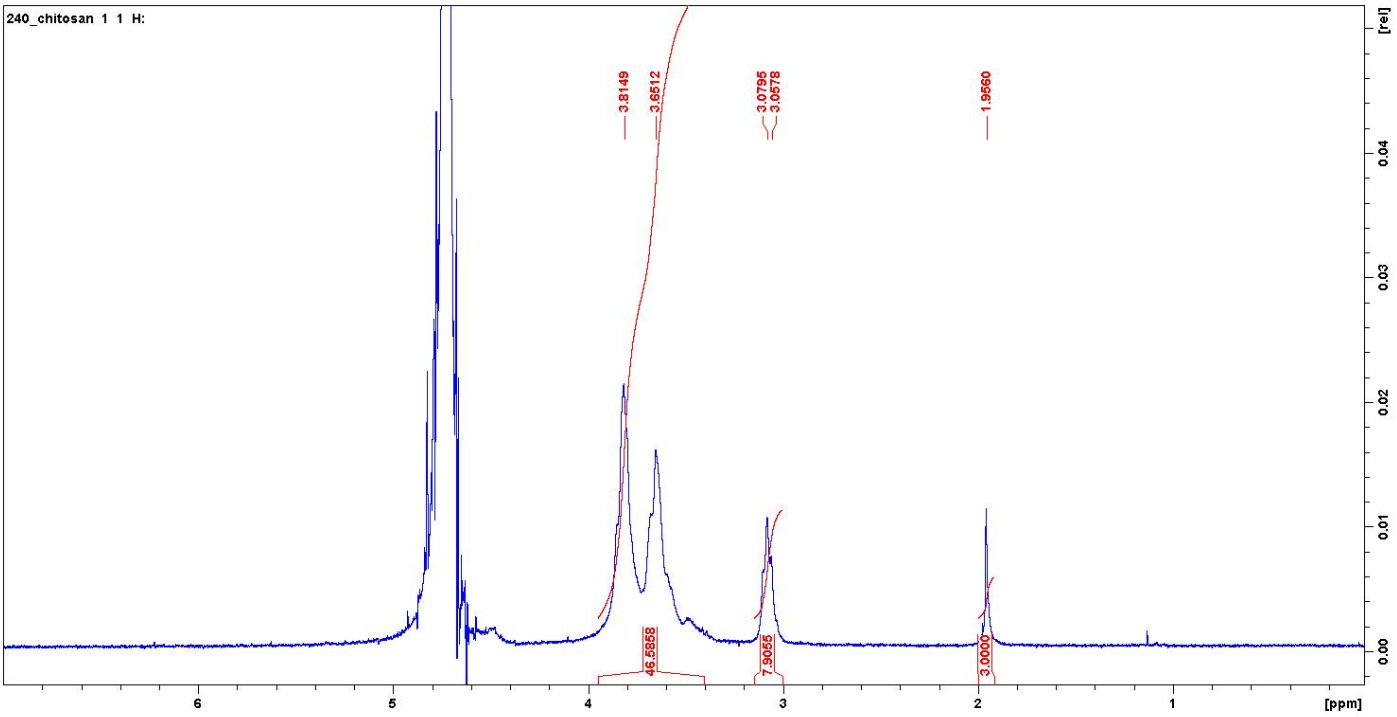

Figure S7. ^{1}HNMR of Chitosan.

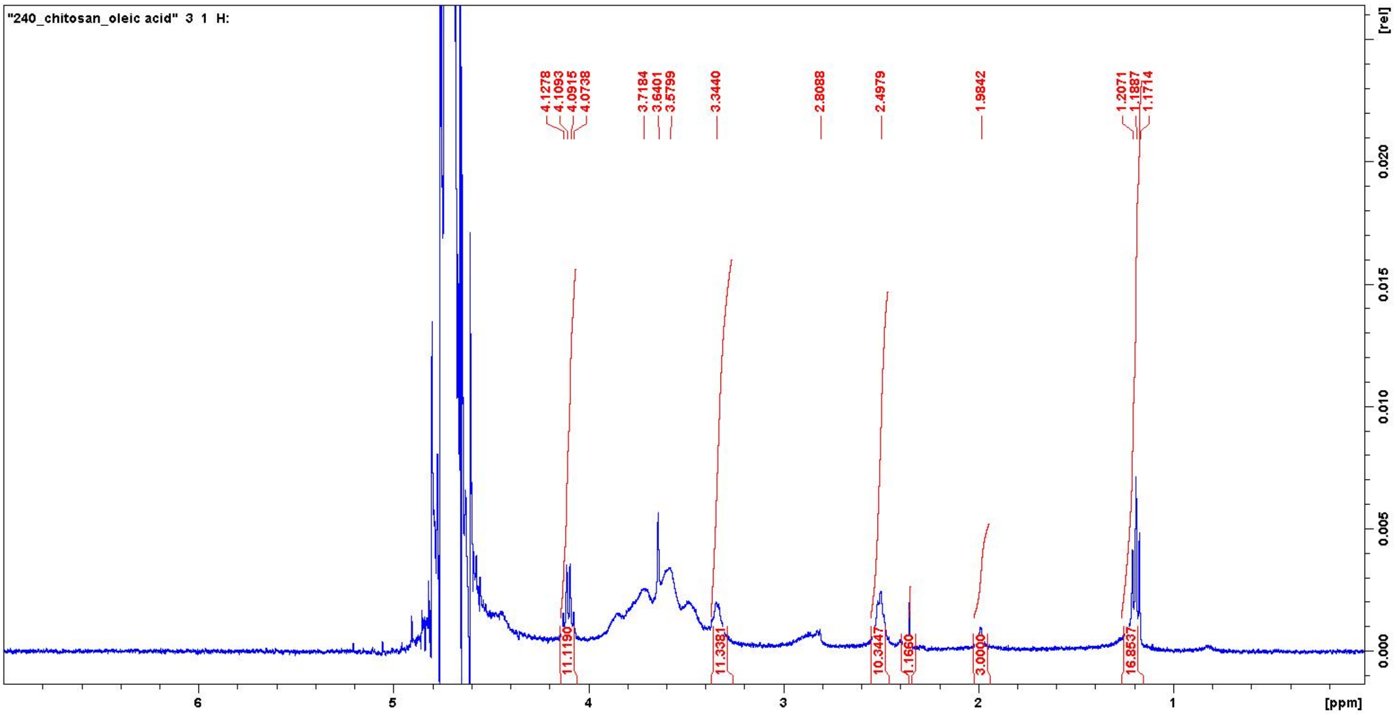

Figure S8. ^{1}HNMR of OA-g-CS.

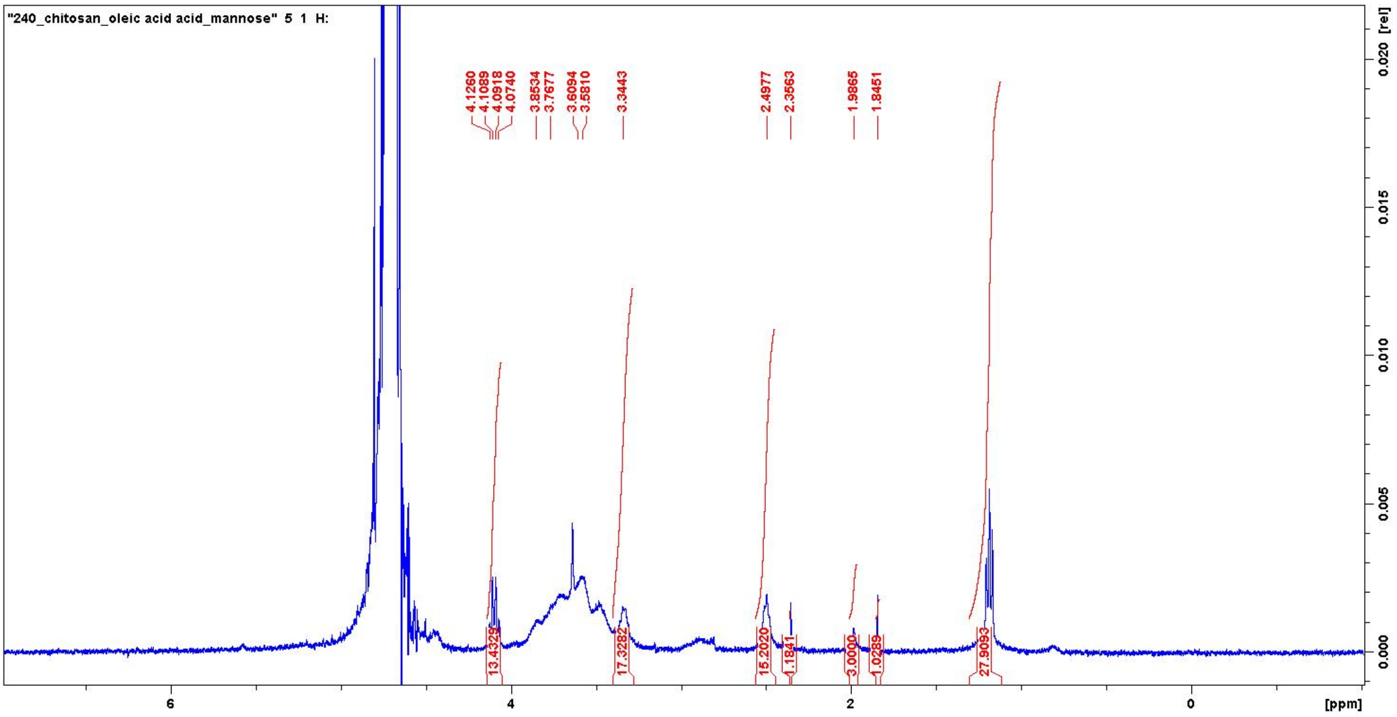

Figure S9. ^{1}HNMR of OA-g-CS-Man.

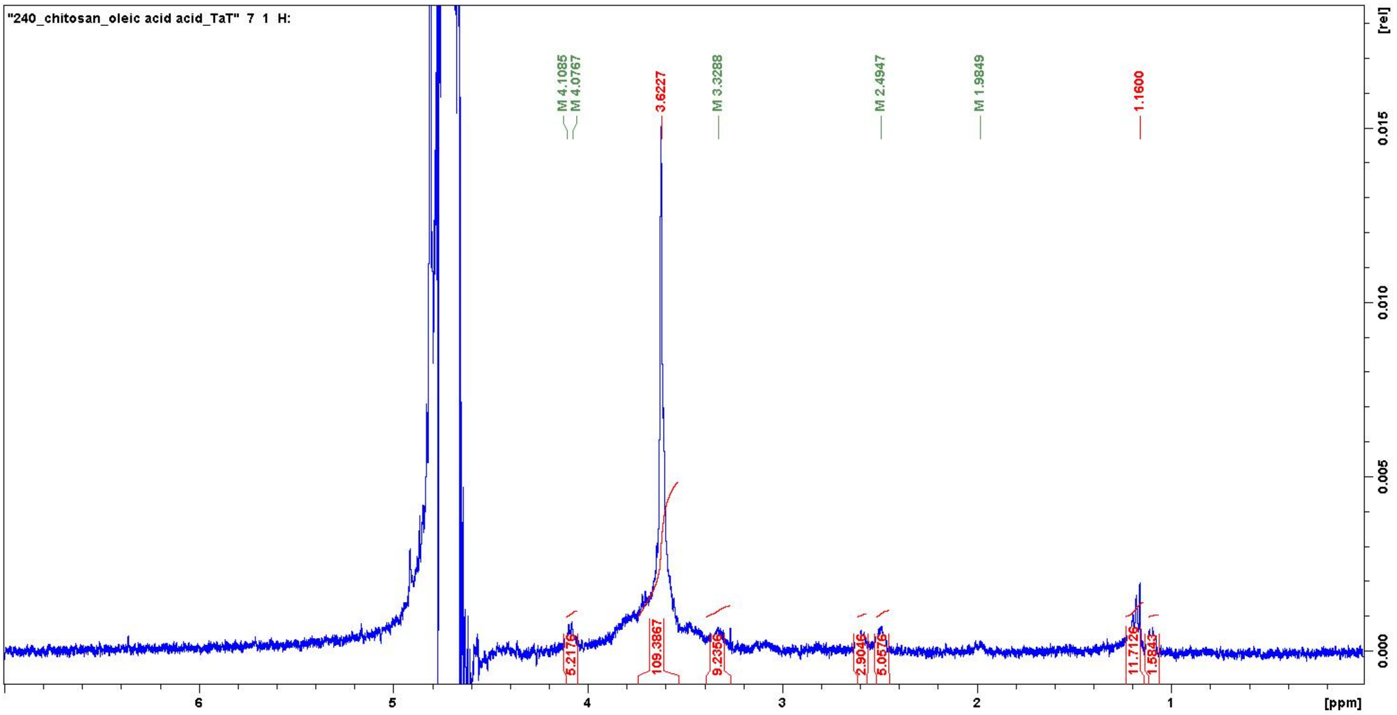

Figure S10. ^{1}HNMR of OA-g-CS-Tat.

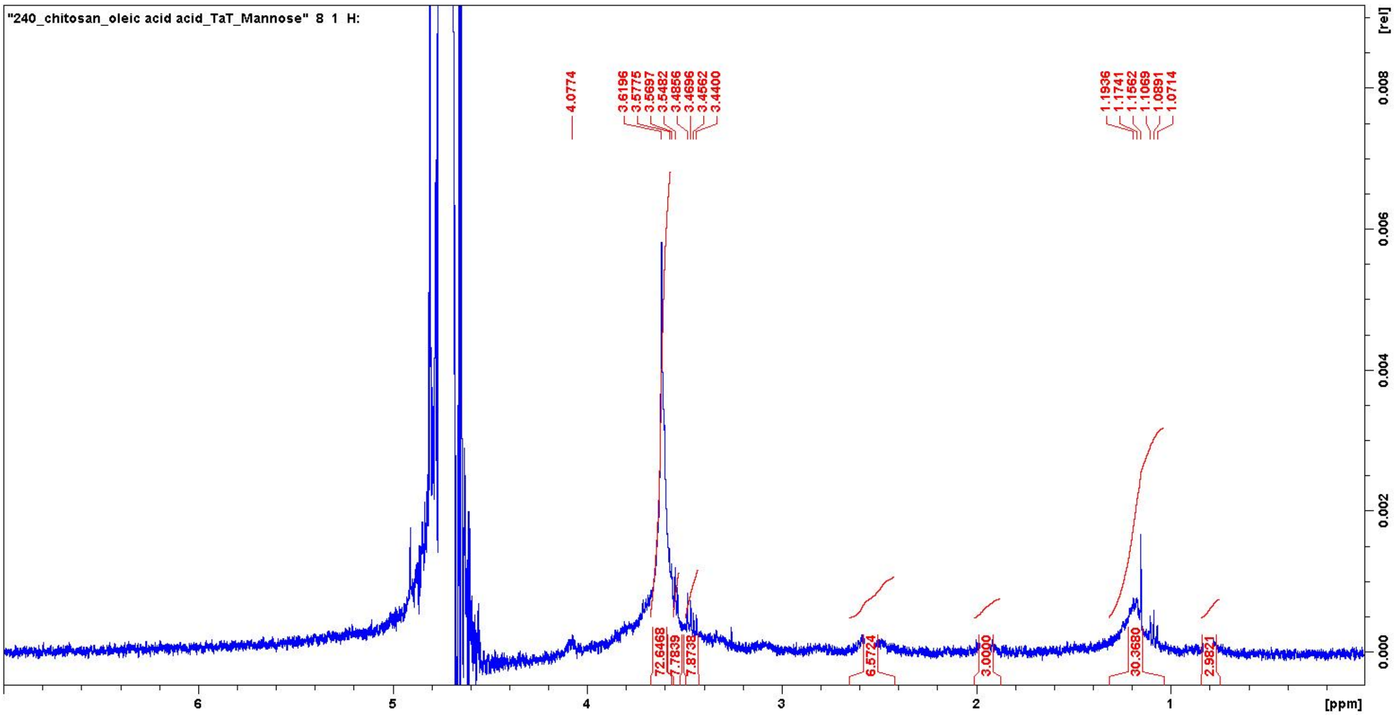

Figure S11. ^{1}HNMR of OA-g-CS-Man-Tat.

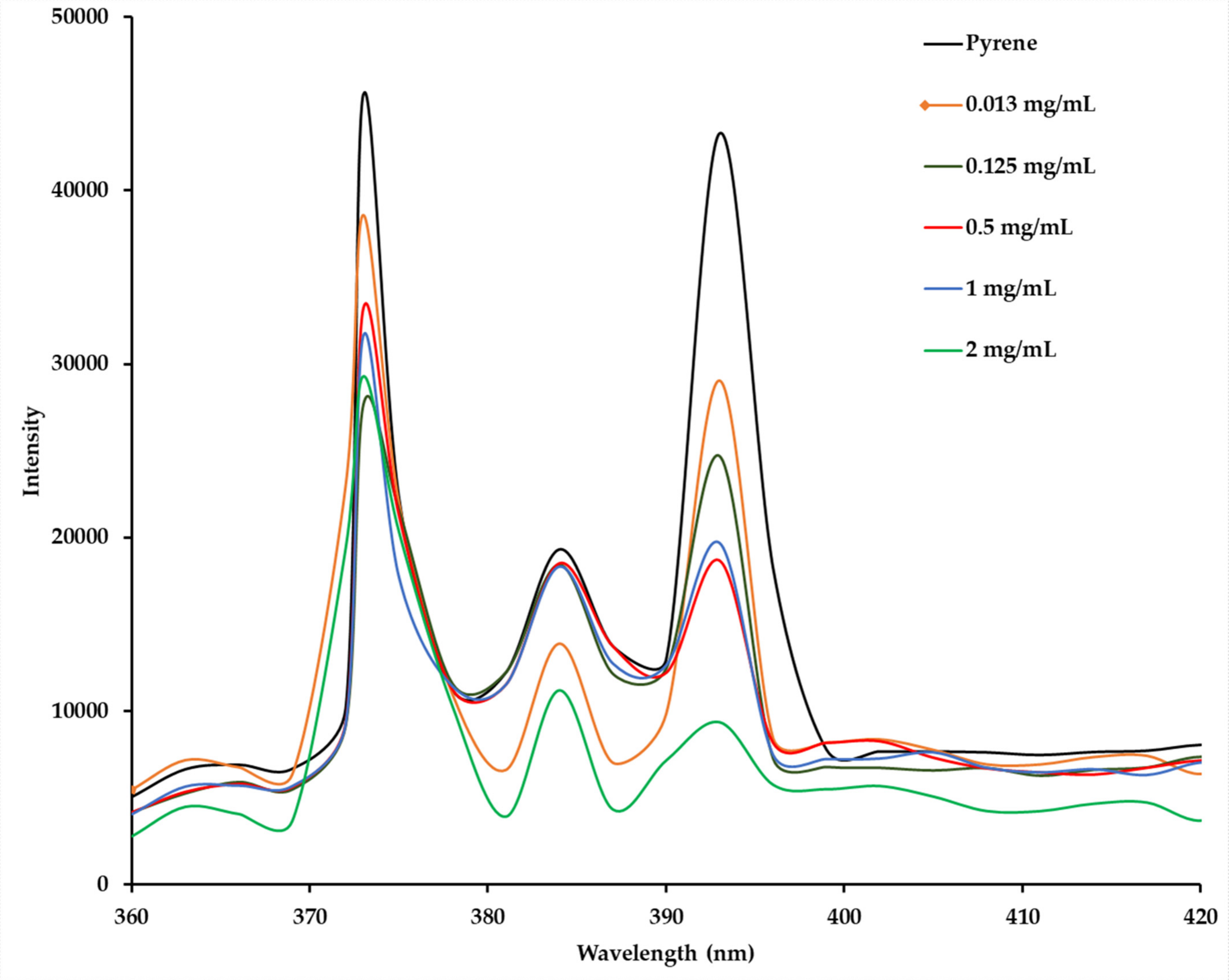

Figure S12. Fluorescence spectra of hydrophobic probe pyrene with increasing concentration of CS-g-OA.
